# Grief-Related Psychopathology from Complicated Grief to DSM-5-TR Prolonged Grief Disorder: A Systematic Review of Biochemical Findings

**DOI:** 10.3390/ijms262411835

**Published:** 2025-12-08

**Authors:** Virginia Pedrinelli, Berenice Rimoldi, Lorenzo Conti, Andrea Bordacchini, Livia Parrini, Laura Betti, Gino Giannaccini, Valerio Dell’Oste, Claudia Carmassi

**Affiliations:** 1 Department of Clinical and Experimental Medicine, University of Pisa, 56126 Pisa, Italy; virginiapedrinelli@gmail.com (V.P.); valerio.delloste@gmail.com (V.D.); 2 Department of Mental Health and Addiction, Azienda USL Toscana Nord-Ovest, 57100 Livorno, Italy; 3 Department of Pharmacy, University of Pisa, 56126 Pisa, Italy; 4 Department of Mental Health and Addiction, Azienda USL Toscana Nord-Ovest, 55100 Lucca, Italy

**Keywords:** prolonged grief disorder, grief, complicated grief, bereavement, neurobiology, peripheral biomarkers, psychoneuroimmunology

## Abstract

Prolonged Grief Disorder (PGD) is marked by enduring and disruptive grief symptoms following the death of a significant other. Although PGD has been recognized as a distinct psychopathological entity within the trauma dimension in the DSM-5-TR, its neurobiological underpinnings remain not fully defined. A systematic literature review was conducted up to September 2025 following PRISMA 2020 guidelines. PubMed, Scopus, Embase and Web of Science were searched using a comprehensive strategy combining MeSH terms and free-text keywords. Eligible studies included human participants, validated grief assessment tools and biomarker assessments. Out of 2140 initial records, 12 studies published between 1989 and 2022 met inclusion criteria. Investigated neuro–psycho–endocrine systems included the hypothalamic–pituitary–adrenal (HPA) axis, catecholamines, oxytocin, endocannabinoids and immune/inflammatory markers. Key findings in pathological grief reactions included altered cortisol rhythms, elevated oxytocin levels, increased pro-inflammatory cytokines and immune system dysregulation. Results are limited by heterogeneity in study designs, small sample sizes, inconsistent use of diagnostic criteria prior to DSM-5-TR and lack of control for psychiatric comorbidities. This review highlights emerging biological correlates of PGD, particularly those involving the stress response, reward-attachment networks and immune/inflammatory pathways. Further standardized, longitudinal research is essential to gain a more defined picture of PGD, to clarify causal mechanisms and to guide targeted therapeutic interventions.

## 1. Introduction

Prolonged Grief Disorder (PGD), previously defined as Complicated Grief or Traumatic Grief, is a recently recognized disorder included as new nosographic entity in the “Trauma and Stressor-Related Disorders” chapter of the latest text-revision of the 5th edition of the Diagnostic and Statistical Manual of Mental Disorders [1]. PGD is a clinical condition identifying individuals in which the loss of a significant one is related to the onset of psychopathological symptoms that persist beyond a certain length of time post-loss and are accompanied by functional impairment. Most people go through a painful but natural grieving process where the intensity of grief-related distress decreases gradually over time [2]. However, research has shown that, in a subset of cases, the grieving process is complicated [3,4]. This complication results in bereaved individuals manifesting exceptionally intense and severe grief reactions that persist abnormally and become progressively debilitating, causing significant health issues, distress and functional impairment, even long after the loss of the loved one [3]. Over the years, several proposals have been formulated for a disorder characterized by such grief reactions, variably termed as Complicated Grief [5], Complicated Grief Disorder [6], Traumatic, Pathological, or Unresolved Grief [7] and Persistent Complex Bereavement Disorder [8].

Currently, a diagnosis of PGD can be set if a person experiences a persistent grief response greater than or equal to 12 months post-loss, with core symptoms including intense, persistent yearning and preoccupation with the deceased, plus several accompanying symptoms, encompassing sadness, anger, identity disruption, emotional numbness and sense of disbelief about the death [1]. Additional symptoms may include guilt, blame, difficulty accepting the loss, an inability to experience positive emotions, loneliness, avoidance, feeling like life is meaningless and bitterness. PGD extends the period of acute grief, interferes with social and occupational functioning and increases risk for a wide range of negative health outcomes.

Clinical studies have shown evidence of its distinction from other psychopathological processes that may occur in the aftermath of a significant loss, particularly Major Depressive Disorder (MDD) and Post-Traumatic Stress Disorder (PTSD) [9,10]. PGD can bring a person’s life to a standstill, interfere with social and occupational functioning and trigger feelings of hopelessness as well as suicidal ideation [10,11].

Regarding the prevalence of PGD, a recent review on the issue found a pooled prevalence of 9.8% after non-violent losses [12]. Rosner and colleagues in 2021 assessed PGD prevalence, utilizing the PGD DSM-5-TR criteria within a representative Western general population cohort: the rate of PGD development among bereaved individuals was determined to be 3.3% [13].

Djelantik et al. (2020) [14] investigated PGD prevalence following highly traumatic forms of loss, such as killings, suicides, disasters, or accidents. Their analysis revealed a random-effects pooled prevalence of 49%, which strongly suggests that this specific characteristic of loss constitutes a significant risk factor for PGD. Elevated PGD prevalence was correlated with the loss of an only child, incidents of violent killing and among people within non-Western settings. Conversely, reduced PGD prevalence rates were found to be associated with longer elapsed periods since the loss and bereavement resulting from a natural disaster, suggesting the necessity of screening for PGD among individuals impacted by traumatic loss and bereavement [14].

Cross-country variance in PGD prevalence rates was explored by a recent meta-analysis, which examined 34 distinct samples from 16 countries: a mean prevalence of approximately 13% was obtained. Notably, this meta-analysis also highlighted that PGD prevalence was higher in countries with more elevated coping capacities, greater capacity to manage or adapt to major challenges and disasters as well as older average population age. This work suggests the relevant influence on PGD prevalence exerted by sociocultural factors and the quality of medical and diagnostic tools utilized across various nations [15].

Despite the increasing number of clinical studies on the neurobiological bases of PGD, results remain fragmentary. Earlier biological hypotheses of atypical grief responses sought to differentiate neuroendocrine and neurochemical changes from those reported in mood disorders and Post-Traumatic Stress Disorder (PTSD). More recently, investigations overall focused on the nature of the stressor involved: while diverse events, not only bereavement, trigger PTSD and depression, PGD is merely restricted to experiences of profound loss and bereavement. Since 2022, the availability of validated psychometric scales has enabled an improvement in diagnostic tools for PGD, which, in turn, has stimulated more detailed research into the psycho-neuroendocrinology and psychoneuroimmunology patterns, which possibly characterize this debilitating mental condition. Furthermore, the diverse clinical manifestations of PGD have revealed the necessity of supplementary validation for both accurate diagnosis and effective patient monitoring. This requirement represents a main challenge that may be attained also through the deployment of appropriate molecular biomarkers. Essentially, the neurobiological bases of PGD are currently believed to be centered on unresolved and persistent adaptive processes. These processes involve a comprehensive array of dimensions, including neurocognitive, emotional, neuroendocrine, behavioral, and even somatic aspects [16]. Compared to non-bereaved controls, subjects experiencing loss showed increased activation in limbic/hippocampal circuits and the basal ganglia—neural areas central to learning, memory and emotion [16,17]. Additionally, the bereavement process has been found to impact fronto-cortical regions responsible for cognitive and executive control [17,18]. It might therefore be assumed that, in PGD, activated brain functions after loss may remain as such or, in the longer term, may even change substantially and persistently. As well, studies of neurobiological correlates of pathological grief reactions hypothesized that the onset and maintenance of symptoms might engage the neural reward system’s activities associated with thoughts of the deceased person [19]. The reward system mediates addictive behaviors through dopaminergic pathways that originate in the midbrain and project to limbo-cortical regions, neural networks known to modulate the motivational salience of stimuli [19,20]. Evidence suggests that activation of this system attenuates nociceptive processing, presumably through the release of endorphins, a class of endogenous opioids [21,22]. This system is posited to bolster individual resilience following the loss of a beloved by facilitating the restoration of wellness after such an emotional “storm,” a function likely altered and compromised in correlation with increased grief severity and duration. Furthermore, reward-related signaling is closely associated with attachment behaviors mediated by oxytocin (OT), a key neuropeptide hormone in modulating social and affective affiliation [23,24]. Dopaminergic, oxytocinergic and opioid receptor systems converge within the *nucleus accumbens*, a central hub of the reward circuitry, enabling the integration of extrinsic and intrinsic reward signals and supporting the establishment of social bonding [19,20,21,22,23,24]. If affective relationships and love activate and promote attachment in the midbrain and neocortex areas via stability signals (as dopamine, nonapeptides and opioids), the disruption of these bonds by loss provokes a series of significant neurochemical changes, by varying both metabolism and clearance of OT and other related molecules, under a limited duration over time for attaining resilience [23]. These mechanisms would prolong differentially in PGD. In substance, grief includes an attachment-specific stress reaction driven by loss including the dopamine system, the opioid system and the oxytocin system [18,19,20,21,22,23,24]. Dopamine plays a chief role in the motivational drive to pursue rewards, while endogenous opioids are more directly implicated in the hedonic experience of reward attainment and, beyond their role in reward processing, are also released in a range of affiliative social contexts, highlighting their broader function in social bonding. Oxytocin, traditionally recognized as a neurohormone essential for parturition and lactation in mammals, has in humans been further associated with psychosocial processes, including reduction of anxiety during social stress and the enhancement of trust [23,24].

Therefore, all these mechanisms would prolong differentially or change relevantly in PGD. Some preliminary results appear encouraging in this regard: the loss of social bonds has been associated with significant changes in OT signaling [25].

Grief has also been shown to shape the complex physiological stress response, including the “fight-or-flight” autonomic response, evoking a general stress-related loss reaction defined by increased plasma catecholamines and heart rate along with other cardiovascular effects while activating the hypothalamic–pituitary–adrenal (HPA) axis and cortisol release [26]. The stress response is a fundamental physiological adaptive response in mammals related to the concept of allostasis or the endogenous adaptive and predictive capacity to cope with stress through time-limited changes in homeostatic set points. Essentially, the early “fight-or-flight” and the relatively delayed HPA responses have evolved in mammals to deal with stressful events through a process of modification and significant variation of specific physiological parameters for a limited time in order to ultimately maintain homeostasis: the preservation of a constant endogenous “milieu” through appropriate adaptive changes [27,28]. Allostasis processes involve sympathetic and neuroendocrine branches intertwined with the activity of the immune system [27,28,29,30,31,32]. Most studies investigating the neurobiology of social loss have focused on the HPA axis (Corticotropin-Releasing Hormone, Adrenocorticotropic Hormone, cortisol) or the S-adenosylmethionine (SAM) methyl donor-related hormones, such as the catecholamines epinephrine and norepinephrine, as primary targets [33,34,35]. Neuroendocrine dysregulation in bereavement has been shown to be influenced by both the proximity and the subjective severity of the loss. Findings indicate that a closer relationship with the deceased and a more intense perception of loss predict a more pronounced endocrine variance [36]. For instance, higher levels of grief and lower social support were associated with higher cortisol levels [37].

Bereavement and catecholamine levels have been related by Jacobs et al., who investigated 24 h urinary free epinephrine and norepinephrine amounts on three successive days in 59 bereaved and anticipatory-bereaved subjects and found higher catecholamine outputs in the bereaved compared to the anticipatory bereaved, reporting, however, not significant differences [38].

The stress response is intersected with the immune and inflammatory response. Therefore, altered immune and inflammatory parameters are supposed to be present in PGD. Some research on immune functioning during bereavement has been carried out over the years. A recent review of associations between grief and biomarkers of immune function reported that most studies on the topic have found significant associations between grief and maladaptive changes in immune parameters in adults, with conflicting results on grief-related changes in cellular immunity [39]. Some studies showed bereaved people reporting higher levels of systemic inflammation, as well as dysregulated immune cell gene expression and a decreased antibody response to vaccination compared with non-bereaved controls. Individual variances in psychological response to bereavement seemed to affect associations between grief responses and immune function [39]. Interestingly, the stress response has been also linked to the activation of reward system pathways as a kind of counter modulatory feedback mechanism [40,41,42]. In line with this relationship between the stress response, reward system, immunity and inflammation, a study reported impaired cortisol and β-endorphin levels alongside altered lymphocytes activation in subgroups of bereaved subjects [43].

Despite a body of evidence with respect to the involvement of the above-mentioned brain areas, systems and mechanisms in bereavement, the lack of an official diagnosis for prolonged grief reactions over the years has overall contributed to ambiguous results related to neurobiological correlates of PGD, essentially because of the different methods applied and time delayed from the grief event investigated. Consequently, it appears quite relevant to appraise a pointed state of the art of the measure of biological markers in the context of the neuroendocrine response to loss, coping and pathological grief response as a solid starting point to achieve more specific targets in this field.

The aim of the present work was therefore to summarize the existing literature by systematically reviewing studies investigating neurobiological mechanisms in pathological grief reactions as a starting point to achieve more specific targets in this field.

## 2. Methods

### 2.1. Literature Search

The present review was developed in accordance with the PRISMA (Preferred Reporting Items for Systematic Reviews and Meta-Analyses) 2020 guidelines. A systematic literature search of studies was performed across PubMed, Scopus, Embase and Web of Science electronic databases, covering studies published up to September 2025.

Since PGD is a relatively new diagnostic entity and it was only included in the Diagnostic and Statistical Manual of Mental Disorders in 2022, for the sake of exhaustiveness, we also included in our research studies published prior to this date, in which pathological grief reactions were assessed through validated psychometric scales. Our search strategy employed the most recent keyword, “Prolonged Grief Disorder” or “PGD,” but also incorporated historical terms commonly used before 2022, such as “Pathological Grief,” “Complicated grief,” or “Traumatic Grief,” to ensure a wider coverage.

In more detail, the search combined controlled vocabulary and free-text terms without filters, restriction, or limits to identify all potentially eligible records: in PubMed ((“persistent complex bereavement disorder” [Title/Abstract] OR “bereavement” [MeSH Terms] OR “grief” [MeSH Terms] OR “Prolonged Grief Disorder” [MeSH Terms] OR “traumatic grief” [Title/Abstract] OR “complicated grief” [Title/Abstract] OR “pathological grief” [Title/Abstract] OR “unresolved grief” [Title/Abstract]) AND (cortisol [Title/Abstract] OR immunity [MeSH Terms] OR “immune response” [Title/Abstract] OR “Neurotransmitter Agents” [Mesh Terms] OR neuroendocrine [Title/Abstract] OR biomarkers [MeSH Terms] OR “Biochemical Phenomena” [Mesh Terms] OR inflammation [MeSH Terms] OR “amino acid metabolism” [Title/Abstract] OR “Tryptophan” [Mesh Terms] OR serotonin [MeSH Terms] OR oxytocin [MeSH Terms] OR “Endocannabinoids” [Mesh])); in Scopus TITLE-ABS-KEY ((“persistent complex bereavement disorder” OR bereavement OR grief OR “prolonged grief disorder” OR “traumatic grief” OR “complicated grief” OR “pathological grief” OR “unresolved grief”) AND (cortisol OR immunity OR “immune response” OR neurotransmitters OR neuroendocrine OR biomarkers OR biochemical OR inflammation OR “amino acid metabolism” OR “tryptophan” OR serotonin OR oxytocin OR endocannabinoids)); and in Embase (‘persistent complex bereavement disorder’:ab,ti OR ‘bereavement’/exp OR ‘grief’/exp OR ‘prolonged grief disorder’:ab,ti OR ‘traumatic grief’:ab,ti OR ‘complicated grief’:ab,ti OR ‘pathological grief’:ab,ti OR ‘unresolved grief’:ab,ti) AND (‘cortisol’/exp OR ‘immunity’/exp OR ‘immune response’:ab,ti OR ‘neurotransmitters’/exp OR ‘neuroendocrine’:ab,ti OR ‘biomarker’/exp OR ‘biochemical’:ab,ti OR ‘inflammation’/exp OR ‘amino acid metabolism’:ab,ti OR ‘tryptophan’:ab,ti OR ‘serotonin’/exp OR ‘oxytocin’/exp OR ‘endocannabinoids’:ab,ti); and in Web of Sciences TS = (“persistent complex bereavement disorder” OR “bereavement” OR “grief” OR “Prolonged Grief Disorder” OR “traumatic grief” OR “complicated grief” OR “pathological grief” OR “unresolved grief”) AND TS = (cortisol OR immunity OR “immune response” OR “Neurotransmitter Agents” OR “neuroendocrine” OR “biomarkers” OR “Biochemical Phenomena” OR “inflammation” OR “amino acid metabolism” OR “tryptophan” OR “serotonin” OR “oxytocin” OR “endocannabinoids”).

### 2.2. Eligibility and Exclusion Criteria

The final selection of the articles was carried out according to the following criteria for inclusion:

1. Human studies.
2. Studies assessing grief reactions by means of validated psychometric scales.
3. Studies that investigated biomarkers associated with bereavement in any kind of biological matrices, plasma, serum, saliva, blood cells and urine to ensure selection of a wider data collection.
4. Articles available in the English language.

Conversely, articles were excluded based on the following criteria:

1. Articles presenting data derived from animal models.
2. Preprints and publications in the form of abstracts, reviews and editorials.

### 2.3. Screening and Selection Process

Two independent reviewers (L.C., V.P.) selected articles for inclusion, further limiting the search to original, peer-reviewed research articles with available full text. It was also decided to examine and screen the reference lists of the selected articles to identify other potentially eligible studies. Any discrepancies were resolved through discussion with a third author (C.C.). In accordance with PRISMA 2020 guidelines, by this approach, the initial search through the primary 4 databases resulted in a total of 2140 records. Following the first screening procedure, 1780 articles were removed at the title review stage due to being duplicates (N = 566) or irrelevant (N = 1214). Subsequently, the 360 selected articles were further screened based on the content of their abstract. At this stage, a total of 177 articles were eliminated, mainly due to irrelevance (N = 157) or inclusion in other publication categories (N = 20).

The remaining 183 articles were further assessed for full-text eligibility. Of these, 171 were excluded for not meeting the inclusion criteria (N = 83), for lack of full-text availability (N = 36), or for being classified as other publication types (N = 52). Of the 83 articles excluded from full-text review, the primary reasons included lack of original human data (N = 6), absence of validated grief psychometric scales (N = 32) and failure to investigate biochemical markers associated with bereavement and grief or assessment of other parameters (N = 45).

**Figure 1:**
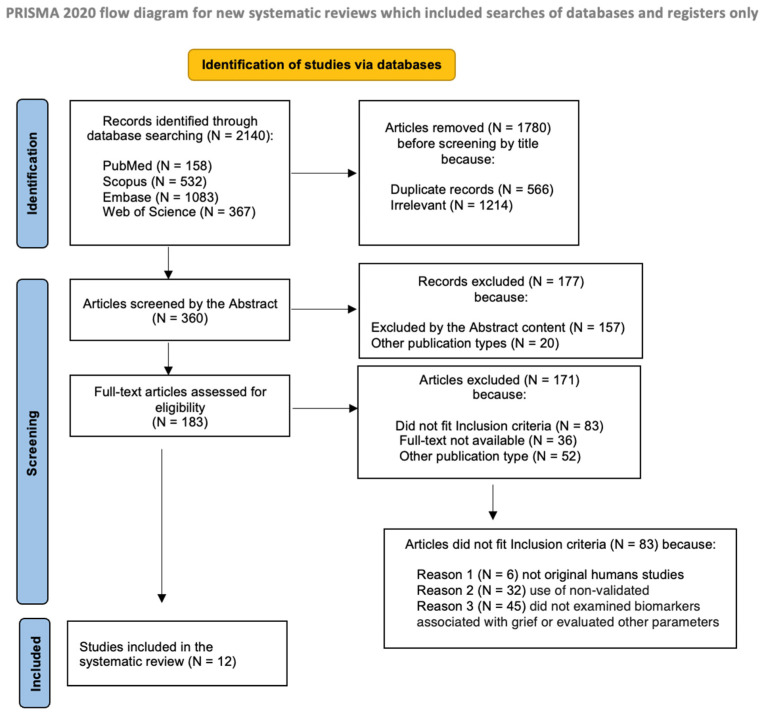
The Preferred Reporting Items for Systematic reviews and Meta-Analyses (PRISMA) flow diagram of the literature search and study selection process.

At the end of these selection procedures, 12 papers remained. Manual screening of the bibliographic lists of these articles was then performed, including also references present in reviews and meta-analyses, but no additional eligible studies were identified. The literature search and the study selection process are summarized in a flow chart, according to the PRISMA 2020 recommendations [44] (see Figure 1). The degree of agreement between the independent authors was good during the whole procedure, and all authors approved the final selected articles.

### 2.4. Quality Assessment

The quality of the papers included was measured by means of a standardized tool adapted from Murad et al. (2018) [45]. Furthermore, we used the Quality Assessment Tool for Observational Cohort and Cross-Sectional Studies (QATOCCSS) to assess the quality of the other type of studies. This tool includes 14 main criteria (with 18 sub-items) that assess critical aspects of internal validity, such as participant recruitment, statistical power, measurement of exposure and outcome variables and control of confounding factors. Each criterion was rated as met, not met, indeterminate, not applicable, or not reported. Any discrepancies between reviewers were discussed until a consensus was reached. Studies were classified into three quality categories based on their risk of bias:

“*Good*”: assigned to studies that fulfilled more than two-thirds of the criteria, indicating a low risk of bias.

“*Fair*”: given to studies that met at least half of the criteria, reflecting a moderate risk of bias but still maintaining result validity.

“*Poor*”: applied to studies that satisfied less than half of the criteria, signifying a high risk of bias that could compromise the reliability of the findings (see Figure 1).

The quality assessment was performed by two independent reviewers (V.P., L.C.), and a third reviewer (C.C.) cross-checked the quality assessment results. Disagreements were discussed and resolved with the research team. The degree of agreement between the independent authors was good.

## 3. Results

Twelve studies were finally selected and included in this review, according to the established criteria. Details of each of the included studies dealing with the appraisal of stress response biological parameters (HPA axis—cortisol and catecholamines) are summarized in Table 1, whilst those papers evaluating other neurobiological markers (oxytocin, endocannabinoids and immune/inflammatory players) are shown in Table 2.

### 3.1. Study Characteristics

The selected studies were published in the period from 1989 to 2022: in 1989, 2021 and 2022, a single article was found; in the period of 2012–2013, four other articles were found, whilst five were identified in the years 2019–2020. Of the 12 studies included in the review, 3 were clinical trials (with a randomized design), 9 had an observational study design, among which were 4 cross-sectional studies, 4 case-control studies and 1 repeated cross-sectional study.

### 3.2. Sample Characteristics

Among the studies investigated, most of the examined samples included both genders, with a prevalence of females across most of the studies. One study included only bereaved females [46], while another did not provide information about the sex characteristics of the sample [52].

All studies evaluated were conducted on samples of adult individuals, except for one conducted on a population of children who had suffered parental loss [48].

Regarding loss characteristics and type of grief, four studies (N = 4, 33.3%) specifically included samples of bereaved spouses [51,52,53,57], one study included women who had experienced the loss of a mother or sister to breast cancer [46] and another included parentally bereaved children [48]. However, in half of the studies considered (N = 6), the sample consisted of subjects who had experienced in a more generic way bereavement due to the loss of a significant one.

The time since bereavement considered in most of the studies (N = 8, 66.7%) was more than one year (ranging from one to five years); however, four studies included subjects who had suffered the loss in the past months before the assessment (on average from three months to twelve months earlier).

Participants reported a mean age of 60.8 years.

Regarding the medication status of the patients, eight studies (66.7%) reported information on this [46,50,51,52,54,55,56,57]. Particularly, one study [52] excluded subjects using psychotropic medications initiated since the death event, and two papers [46,54] excluded subjects using anti-depressant drugs. The remaining studies included subjects using psychotropic or other drugs in varying percentages.

Using the NIH QATOCCSS tool, eight studies were rated as *Good* and four as *Fair*. No studies were rated as *Poor*. Common methodological shortcomings included small sample sizes (sometimes fewer than 30 participants), reliance on cross-sectional designs that preclude causal inference, variability in pharmacological treatment status across participants, lack of follow-up in several studies and heterogeneity in both type of loss and time since bereavement.

### 3.3. Assessments

#### 3.3.1. Grief Assessment

Since screened papers were published in a wide period encompassing papers published before the DSM-5-R PGD criteria, the psychometric assessment scales were relatively variable over time. The commonest grief measurement (used in nine studies) was the Inventory of Complicated Grief (ICG) [58,59]. For the remaining four papers, the scales used for pathological grief assessment included the Grief Experience Inventory (GEI) [60], the Structured Clinical Interview for Complicated Grief (SCI-CG) [61], the Inventory of Complicated-Grief-Revised (ICG-R) [62] and the Prolonged Grief Disorder-13 (PG-13) [3].

#### 3.3.2. Psychiatric Comorbidity

Seven of the twelve papers included in this review variously reported comorbidities with pathological grief reactions. The most frequently occurring comorbidity was that with depressive disorders and depressive symptoms, which was reported in seven studies [49,51,53,55,56,57]. Studies considered also co-occurring post-traumatic stress symptoms [48] and anxiety symptoms [48,55]. Finally, one study evaluated comorbidity with schizophrenic and affective psychosis [51].

Two papers considered the presence of a current major psychiatric disorder as an exclusion criterion [46,52], whilst five studies did not report information about psychiatric comorbidities.

### 3.4. Biomarkers

#### 3.4.1. Biological Matrices

Papers dealing with the appraisal of the targeted biological parameters in PGD were screened without specifying the biological fluid investigated. This permitted us to eventually include all biological matrices such as whole blood, blood cells, plasma, serum, saliva and urine. The search yielded articles meeting established criteria which investigated plasma, serum, whole blood cell culture supernatants and saliva. None of them examined urine samples. Specifically, four studies (33.3%) analyzed biomarkers in the serum [51,55,56,57], three in the plasma (25%) [46,52,54], one examined supernatant of mitogen-activated whole blood cell cultures together serum (8.3%) [51], while another one explored blood cells only (8.3%) [53]. Four studies investigated biomarkers in salivary samples (33.3%) [46,48,49,50].

#### 3.4.2. HPA Axis: Cortisol

Five of the twelve selected studies were focused on the appraise of plasma, serum, or salivary cortisol levels. One study compared salivary cortisol outputs in women with Complicated Grief (CG Group) to that in bereaved women without Complicated Grief (NCG Group) to determine whether these two groups differed in the slope of their cortisol outputs across morning, afternoon and evening time points [46]. Results demonstrated that those with Complicated Grief had a cortisol flatter slope across the day, accounting for education and body mass index. More specifically, cortisol was lower at 45 min post-wake and higher at 4 pm in the CG group than in the NCG group.

Another selected survey measured morning and evening salivary cortisol levels in 32 depressed non-bereaved individuals, 15 bereaved individuals without elevated PGD symptoms and 9 depressed bereaved individuals with elevated PGD symptoms [50]. Noteworthy, no significant differences in waking cortisol levels between the two bereaved groups was found, but depressed bereaved individuals without elevated PGD symptoms had significantly lower levels of log-cortisol at wake and flatter diurnal slopes compared to depressed non-bereaved subjects, suggesting that those who were bereaved had more dysregulated cortisol patterns, even if PGD symptomatology seemed to have little effect.

The cortisol awakening response (CAR) was the exclusive focus of one investigation. Kaplow et al. [48] measured the CAR in saliva over three consecutive days in 38 children who had recently lost a parent and 28 surviving caregivers. The findings revealed a significant inverse relationship between the child’s CAR attenuation after 1 day and heightened symptoms of maladaptive grief, anxiety, depression, post-traumatic stress and avoidant coping. Furthermore, elevated levels of parental maladaptive grief correlated with a blunting of the child’s CAR. The authors hypothesized that a weakened CAR may reflect the accumulation of allostatic load and/or the influence of emotionally difficult family events and their subsequent processing (or lack thereof), which intensifies stress for grieving children experiencing severe psychological distress.

Bell and colleagues [50] in a randomized clinical trial evaluated within-session changes in salivary cortisol and salivary α-amylase (sAA) for 54 older adults with CG who received a psychotherapy intervention (Accelerated Resolution Therapy, ART), comparing them with perceived stress. The trial compared ART versus a 4-week wait list control condition in relation to changes in CG symptoms. Neither the pre-ART salivary cortisol value nor within-session change in perceived stress levels were associated with pre-to-post-ART change in cortisol value. The variable most strongly associated with change in cortisol was the use of antibiotic/anti-viral medication. Other variables associated with within-session change in cortisol value included use of over-the-counter supplement and ART visit number.

Another study investigated serum cortisol amounts in the context of the appraisal of circulating endocannabinoid levels in pathological grief: Kang et al. [56], in a sample of adults aged 50 years and older, divided in three groups (Grief with High loneliness, Grief with Low loneliness and Healthy Controls), revealed that cortisol concentrations did not differ between them.

#### 3.4.3. Catecholamines

One study [47], which utilized a small sample of 16 bereaved individuals diagnosed with CG, predominantly women, assessed plasma levels of norepinephrine, epinephrine and dopamine both pre- and post-psychotherapeutic treatment. Interestingly, individuals with the highest pre-treatment epinephrine levels also exhibited the highest levels of complicated grief symptoms following treatment. Essentially, only pre-treatment epinephrine levels significantly predicted post-treatment ICG scores (after controlling for pre-treatment ICG scores). Conversely, pre-treatment noradrenaline and dopamine were not significant predictors of post-treatment ICG scores. However, it should be noted that none of the measured catecholamines were found to be unrelated to post-treatment CG symptoms, despite the unique predictive power of epinephrine. Another investigation indirectly appraised the sympathetic branch of the stress response by measuring sAA together with cortisol pre- and post-ART in CG [50]. This work rather highlights more association between adrenergic tonus in subjects affected by CG than cortisol. They also showed, however, an association for sAA, like for cortisol, with confounding variables and drug therapies such as antibiotics and antivirals.

#### 3.4.4. Oxytocin

Only one study was identified that specifically measured oxytocin (OT) levels in subjects presenting an abnormal grief response. Bui and coauthors examined plasma OT amounts in 139 bereaved adults divided in three groups: those with a primary diagnosis of CG; those with a primary diagnosis of MDD; those mentally healthy bereaved controls, (without psychiatric symptoms) [54].

The findings indicated that OT levels were significantly higher in the CG group compared to the MDD group, although no significant difference was found when compared to the bereaved controls. It is noteworthy that, in this study, adjusted regression models confirmed that a primary or probable CG diagnosis was significantly associated with elevated OT levels.

#### 3.4.5. Endocannabinoid System

Two studies investigated peripheral endocannabinoid levels in the context of grief. Kang et al. [56] explored the association between loneliness and serum concentrations of two endocannabinoid compounds, N-arachidonoylethanolamide (Anandamide, AEA) and 2-arachidonoylglycerol (2-AG), in grieving older adults. Their specific aim was to link baseline loneliness and grief symptom trajectories with the levels of these molecules. The study utilized a sample of 64 adults initially divided into Grief and Healthy Control (HC) groups. The Grief group was further stratified into subgroups based on loneliness: Grief with High loneliness (Grief-HL) and Grief with Low loneliness (Grief-LL). Results demonstrated that serum AEA levels were significantly higher in the Grief-HL group compared to HC. However, no significant differences were observed between the Grief-HL and Grief-LL subgroups. Conversely, serum levels of 2-AG and cortisol (discussed previously) showed no significant differences across the three groups.

Harfmann and coauthors [55] investigated the same parameters, serum AEA and 2-AG, reporting similar results to those found by Kang et al. [56] but finding that serum AEA concentrations were positively correlated with depressive and anxiety symptoms only in grievers with low ICG scores (<30), without significant results for serum 2-AG.

#### 3.4.6. Immunity/Inflammatory Factors

Four studies investigating the possible role of immune/inflammatory biomarkers in pathological grief were found among the selected twelve ones. First, the comprehensive work by Pettingale and colleagues [51] focused specifically on altered immune status in pathological grief following conjugal bereavement, examining samples exclusively from bereaved spouses. The authors assessed serum levels of several acute-phase proteins, encompassing Immunoglobulins G, A and M (IgG, IgA, IgM), transferrin, alpha-1 acid glycoprotein, alpha-2 macroglobulin, ceruloplasmin and alpha-1 anti-trypsin. Furthermore, they isolated lymphomonocytes from whole blood to quantify various immune cells: CD3+ total T cells, CD4+ helper/inducer T cells, CD8+ suppressor/cytotoxic T cells and CD16+ lymphocytes (Natural Killer, NK cells). The key finding of this study demonstrated that the severity of grief symptoms was negatively associated with the number of CD16+ NK cells and serum levels of alpha-2 macroglobulin. Additionally, the length of bereavement correlated negatively with the numbers of CD8+ T cells and serum concentrations of IgG and transferrin.

Another investigation [52] assessed a gene *x* environment interaction in bereaved subjects, comparing those with CG, those with non-complicated grief and non-bereaved controls (HC). The researchers first genotyped subjects for Single Nucleotide Polymorphisms (SNPs) related to the proinflammatory interleukins IL-6, IL-1β and Tumor Necrosis Factor-alpha (TNF-α), specifically focusing on the IL-6-172 and -572 variants. Subsequently, they assessed systemic inflammation by measuring circulating plasma markers in bereaved subjects versus married/partnered older adults. Measured markers included IL-6 and the soluble modulatory factors interleukin-1 receptor antagonist (IL-1RA) and soluble tumor necrosis factor receptor II (sTNFRII). Significantly higher circulating levels of IL-1RA and IL-6 were reported in the overall bereaved group compared to non-bereaved controls. Although no differences were observed in other plasma inflammatory factors between the CG and non-complicated grief subgroups, the authors specifically examined the association between inflammatory status in bereaved individuals and the presence of the IL-6-172 polymorphism.

Fagundes et al. [53] aimed to identify if grief was related to the immune activity status in a sample of almost one hundred bereaved spouses. These authors measured the culture supernatants obtained from mitogen-activated whole blood cell cultures of all enrolled individuals to evaluate lymphomonocyte production of cytokines and release of inflammatory factors. Activated lymphomonocytes from bereaved individuals with a higher grief severity released higher levels of the proinflammatory cytokines interferon-γ (IFN-γ), IL-6 and TNF-α in culture supernatants than those with less grief severity. Patients reporting more severe symptoms of depression had higher levels of pro-inflammatory cytokines than those with less severe ones. Authors argued that such inflammatory markers might help to distinguish bereaved individuals based on the severity of their grief, such that those with higher levels of grief had higher levels of inflammation than those with lower ones.

Finally, a paper exploring associations between grief symptoms and IL-6 serum levels as a biomarker of inflammation during acute stress in a sample of over 100 bereaved spouses found that those experiencing high grief symptoms experienced a significantly greater increase in interleukin-6 levels compared to those experiencing lower grief symptoms. The authors suggested that grief levels of recently bereaved people were associated with the rate of change in IL-6 levels following a subsequent stressor, beyond depressive symptoms [57].

## 4. Discussion

In recent years, increasing attention has been devoted to understanding the pathophysiological mechanisms underlying complicated grief, a condition now formally recognized as Prolonged Grief Disorder (PGD) following its inclusion in the DSM-5-TR [1]. Despite a still limited and methodologically heterogeneous body of literature, the present systematic review provides an initial neurobiological map of the alterations potentially involved in the onset and maintenance of PGD, giving suggestions for possible future directions in research and therapy of this invalidating mental condition. Relatively few papers were collected from the present selection of databases addressing the issue, indicating that much remains to be researched on the neurobiology of PGD. The most consistently investigated biomarkers in pathological grief were those related to the HPA axis and inflammatory and immunological responses. Few other studies sporadically examined the sympathetic catecholaminergic circuits, as well as the reward system with oxytocinergic, dopaminergic and endocannabinoid signaling. Among all selected works, HPA axis dysregulation occupied the central role. Cortisol, the principal downstream effector hormone of this whole-body adaptive neuroendocrine axis, has been the focus of several investigations that identified altered diurnal patterns in individuals with prolonged grief.

For example, O’Connor et al. [46] reported an abnormal diurnal cortisol release in bereaved women with complicated grief. This profile was characterized by a flattened slope—marked by lower levels upon awakening and elevated afternoon levels—a pattern consistent with other chronic stress-related disorders. Indeed, such dysregulation has been linked to severe health outcomes, including cardiovascular, metabolic, and immune impairment. Similarly, in pediatric populations, Kaplow et al. [48] described a blunted Cortisol Awakening Response (CAR) in children recently experiencing parental loss, linking this specific dysregulation to maladaptive grief, depressive symptoms and anxiety. These collective findings suggest the presence of HPA allostatic overload in pathological grief. However, the data are not entirely convergent. Holland et al. [49] found no significant differences in morning cortisol levels between bereaved individuals with and without elevated prolonged grief symptoms. Although a flattened diurnal slope was still noted across all bereaved participants, this lack of specificity raises questions regarding HPA alterations as unique markers for complicated grief, distinguishing it from general mood or stress-related disorders. Furthermore, given that HPA axis activity is known to vary with the time elapsed since exposure to stressful events in post-traumatic patients [49], future PGD studies should specifically address the impact of bereavement duration on cortisol release.

Stress-related physiological systems also involve catecholamines, including adrenaline, noradrenaline and dopamine, considered among the main targets in the search of PGD biological correlates. However, among the studies reviewed, only one matched the established eligibility criteria [47]. This study identified a positive association between pre-treatment epinephrine levels and post-treatment PGD symptom severity, while other catecholamines were not significantly associated. This suggests a potentially specific role of adrenergic hyperactivation in sustaining emotional distress, contributing to somatic symptoms often observed in PGD such as sleep disturbances, tachycardia and fatigue.

The work of Bell and coauthors reports association between CG and sAA, a biomarker linked to the autonomic stress response and catecholamine activity [50]. These authors, however, report that both salivary cortisol and amylase are under the influence of concomitant administration of common drugs as antibiotics/antivirals, pointing out the problem of restraining these biases.

Substantially, the present systematic review confirms the involvement of the stress response in PGD but also highlights a more pronounced interest towards the HPA branch rather than the sympathetic one. Instead, these branches are under mutual regulation, and the evaluation of catecholamines remains highly relevant across bereavement, grief and prolonged grief states. Moreover, while norepinephrine and epinephrine principally mediate stress-related sympathetic functioning during coping, dopamine may specifically reflect alterations at the crossroad between the stress response and reward system activity. Consequently, our findings highlight a significant gap in the current literature, suggesting that more comprehensive studies focused equally on all branches and players of the stress response are warranted.

A particularly novel line of research has investigated the role of oxytocin, a neuropeptide associated with social bonding and attachment. The loss of a close attachment figure is believed to activate not only general stress pathways but also neurobiological circuits specific to attachment disruption. In this context, Bui et al. [54] found elevated plasma oxytocin levels in individuals with PGD compared to those diagnosed with MDD, demonstrating the specificity of OT response in PGD vs. mood disorders. This finding supports the hypothesis of a dysregulated and persistent activation of the attachment system in PGD, characterized by an unresolved search for the lost bond. Such findings may account for the relational and treatment-resistant nature of the disorder and open avenues for interventions targeting attachment and social reward circuitry through pharmacological, psychotherapeutic, or rehabilitative approaches.

The endocannabinoid system, recognized for its role in modulating stress and emotional processing, has also been implicated in grief responses. Kang et al. [56] reported elevated levels of AEA in bereaved individuals experiencing high levels of loneliness. While this alteration was not specific to PGD, it suggests that endocannabinoid signaling may be sensitive to the emotional context of social isolation during bereavement, making it a possible target for future interventions. Harfmann and coauthors [55] reported similar results with positive correlations between AEA and depression or anxiety scores, suggesting that these endocannabinoid compounds were rather related to anxiety and depression in grief.

The search for inflammatory and immune biomarkers represents a growing area of interest in PGD. Several studies, notably those by Fagundes et al. [53] and Brown et al. [57], established an association between grief severity and elevated levels of pro-inflammatory cytokines such as IL-6, TNF-α and IFN-γ. This was further supported by findings in the cell culture milieu of stimulated whole blood cells [53], indicating an increased state of blood cell activation in prolonged grief. These results are consistent with stress-related models postulating that chronic activation of the stress response drives low-grade systemic inflammation. Other authors [51] also highlighted reduced activity of NK CD16+ cells and CD8+ lymphocytes in individuals with pathological grief symptoms. This dual alteration implies disturbances at the interplay between immune suppression and inflammation in prolonged grief, together an increased vulnerability to infections and chronic diseases, emphasizing the necessity of medical monitoring for patients with persistent grief symptoms. Intriguingly, one selected paper [52] revealed a gene *x* environment association between the IL-6-172 polymorphism and increased circulating levels of both IL-6 and the modulatory soluble factor IL-1RA in CG, suggesting the activation of specific inflammatory pathways in this condition.

A key aspect to consider for the development of suitable biomarker platforms in PGD is also the type of biological sample examined. Relatively new biotechnological tools called -omics techniques, such as genomics, epigenomics, transcriptomics, proteomics and metabolomics, have revealed the importance of methodological choices for the correct interpretation of results. This concern also applies to studies aimed at identifying targeted and specific biomarkers [63,64,65]. The present review, which focused on studies reporting results for targeted metabolic and proteomic substrates, such as cortisol and cytokines, found that plasma and serum were the predominant biological matrices used. However, saliva and mitogen-activated blood cells were also utilized. Specifically, the assessment of biomarkers related to the activation degree of white blood cells was highlighted for its potential to circumvent biases linked to frequently used sample matrices as serum [53]. Notably, no study included in this review analyzed urine samples, despite this matrix being of recognized interest in the field. This absence likely reflects the common scientific opinion that urine composition is susceptible to numerous variables also related to problematic collection procedures.

On the other hand, urine, as a non-invasive biological sample, can offer useful complementary information when assessed alongside blood-derived matrices, proving particularly useful for evaluating circadian and/or neuroendocrine biomarkers [47,66]. The fact that specific diagnostic tools for PGD are now available encourages the implementation of both proteomic/metabolomic and targeted assessments in multiple biological matrices, including not only serum/plasma but also blood cell culture supernatants, intracellular (e.g., platelets, lymphomonocytes) contents, saliva and urine, enabling the possibility at appraising different components of the same parameters in different body fluids.

The overall quality of the included studies was moderate-to-good (8 rated *Good*, 4 rated *Fair*). However, methodological limitations such as small samples and cross-sectional study design should be considered when interpreting our conclusions.

Although this review contributes a synthesis of the current main underpinnings of peripheral biomarkers in PGD, our findings should be interpreted cautiously. Many of the included studies employed observational designs, had limited sample sizes and relied on non-standardized diagnostic criteria, often due to the absence of a formal PGD diagnosis prior to 2022. These limitations also impact the possibility to address gender and age effects on the investigated biological parameters in our selected studies. Earlier research used terms such as “complicated grief” or “complex persistent bereavement”, confounding data synthesis. Furthermore, psychiatric comorbidities, notably major depression and PTSD, were not always adequately controlled, and the use of psychotropic medication was inconsistently reported, thereby limiting the comparability of results across studies.

## 5. Conclusions

Current evidence on the etiopathogenetic mechanisms and potential biomarkers of PGD is limited and often inconsistent. Although the growing interest in this field of research, further effort is needed to investigate the biochemical correlates of PGD to assess whether there is an interaction between different metabolic systems and pathways. From a clinical perspective, enhancing the understanding of biochemical factors associated with PGD could help clinicians identify individuals at higher risk, facilitate early diagnosis and enable timely treatment. The complex presentation of PGD can be elucidated by underlying biochemical biomarkers considering possible influencing factors such as comorbidities, administered drugs, even non-psychotropic compounds, and post-loss timing. Further standardized, longitudinal research is essential to gain a broader and more defined neurobiological picture of PGD, as well as to clarify causal mechanisms and guide targeted therapeutic interventions.

## Figures and Tables

**Table 1:** Characteristics of the included studies referring to peripheral biomarkers related of the response to stress: HPA axis–cortisol and sympathetic catecholamines.

Study	Year	Country	Quality Rating	Type of Study	Sample Characteristics					Grief Assessment	Measured Markers	Biological Sample	Main Findings
					Grief Types/Groups	Loss Relation	Time Since Loss	N/n per Group	Age (Years, Mean/Range or SD)				
O’Connor et al. [46]	2012	USA	Good	Case-control study	Bereaved women; CG vs. Non-CG	Mother; sister (death to breast cancer)	Up to 5 years post-loss	N = 24, women CG N = 12 Non-CG N = 12	CG = 42.67 (10.54) Non-CG = 46.91 (9.32)	ICG	Cortisol	Salivary (at waking, 45 min post-waking, 4 PM and 9 PM on three consecutive days)	Lower cortisol levels at 45 min post-waking, and higher cortisol levels at 4 PM in CG group vs. Non-CG group—flatter diurnal cortisol rhythms associated with CG.
O’Connor et al. [47]	2013	USA	Good	Randomized clinical trial	Bereaved adult individuals	Parent (44%), spouse (31%), child (6%), sibling (13%), other (e.g., close friend, 6%)	Widely variable: mean 87 months (SD = 123.9), median 38 months	N = 16; females N = 14 (87.5%); males N = 2 (12.5%)	64 (SD = 4.3)	ICG (at study intake—up to 4 weeks before the first therapy session- and at week 20, following the termination of treatment	Catecholamines (norepinephrine, epinephrine, dopamine)	Plasma	Pre-treatment epinephrine levels were predictive of post-treatment ICG scores, taking into account the pre-treatment ICG score, whereas pre-treatment levels of other catecholamines were not. Age was not a significant predictor within the model. When each catecholamine level was used to predict grief symptom levels at post-treatment only, none of the catecholamine levels were able to predict ICG nor BDI-II scores, taking into account pre-treatment BDI-II results.
Kaplow et al. [48]	2013	USA	Good	Randomized clinical trial	Parentally bereaved children (recent parental loss) and their surviving caregivers	Parent	Previous 6 months	N = 66; children N = 38 (20 girls); their surviving caregivers N = 28 (23 women)	Children: 9.6 (SD = 2.04); surviving caregivers: 42.76 (SD = 8.33)	ICG-R in children; PG-13 in surviving caregivers	Cortisol in Awakening Response (CAR)	Salivary (at waking and 30 min post-waking, on three consecutive days)	Significant correlation between attenuation of first-day CAR and increased symptoms of anxiety, depression, post-traumatic stress and avoidant coping strategies. Higher levels of maladaptive grief were associated with attenuation of first-day CAR.
Holland et al. [49]	2020	USA	Fair	Case-control study	Older adults: depressed non-bereaved individuals (Group 1); depressed bereaved individuals without elevated PGD symptoms (Group 2); depressed bereaved individuals with elevated PGD symptoms (Group 3)	Spouse or partner (33%); parent (16.7%); sibling (12.5%), friend (12.5%): child (4.2%). Most of deaths were due to natural causes (87.5%)	On average 3.1 years	N = 56; Group 1 N = 32; Group 2 N = 15; Group 3 N = 9; females N = 34 (60.7%); males N = 22 (39.3%)	69.9 (SD = 7.6); Group 1 70.4 (SD = 7.5); Group 2 69.4 (SD = 7.5); Group 3 68 SD = 7.3)	PG-13	Cortisol	Salivary (at wake, 5 pm and 9 pm across two consecutive days)	Bereaved subjects had significantly lower levels of log-cortisol at wake and dysregulated cortisol rhythm compared to depressed non-bereaved. No significant differences in waking cortisol levels between the two bereaved groups (Groups 2 and 3).
Bell et al. [50]	2020	USA	Good	Randomized clinical trial	Older bereaved adults (aged 60 years or older) with a diagnosis of CG	//	At least 12 months prior to enrollment	N = 54; N = 32 randomly assigned to receive ART (accelerated resolution therapy) psychotherapy intervention immediately; N = 22 assigned to the control condition (4-week waitlist); females N = 42 (84%); males N = 8 (16%)	68 (SD = 6.7); age group by years: less than 65 34%; 65–74 46%; 75 or older 20%; age group by sex: females 67.4; males 71.1	ICG	Cortisol (before and after ART session 1 and 4) and salivary Alfa-Amylase (sAA) (before and after each ART session)	Salivary	The change in pre-ART salivary cortisol level in relation to perceived stress was not associated with the change in cortisol level before and after ART. The variable most strongly associated with cortisol change was antibiotic/antiviral drug use; other associated variables included the use of over-the-counter supplements and the number of ART visits. Conversely, pre-ART sAA was strongly associated with changes in sAA before and after ART. However, consistent with the cortisol findings, the most strongly associated variable was antibiotic/antiviral drug use. Other variables of influence were different medications, months and number of hospital admissions since the loved one’s death.

CG: complicated grief; Non-CG: non-complicated grief; ICG: Interview for Complicated Grief; BDI-II: Beck Depression Inventory-II.

**Table 2:** Characteristics of the included studies referring to peripheral biomarkers of oxytocin and endocannabinoid systems as well as immune/inflammatory response (cytokines and T cell subsets).

Study	Year	Country	Quality Rating	Type of Study	Sample Characteristics					Grief Assessment	Measured Markers	Biological Sample	Main Findings
					Grief Types/Groups	Loss Relation	Time Since Bereavement	N/n per Group	Age (Years, Mean/Range or SD)				
Pettingale et al. [51]	1989	UK	Fair	Cross-sectional study	Bereaved spouses	Spouse	In average 2 years (from one to three and half years)	N = 33; females N = 27; men N = 6	Mean = 63 years	Grief Experience Inventory	IgG, IgA, IgM, transferrin, alpha-I acid glycoprotein, alpha-2 macroglobulin, cerulosplasmin and alpha-1 anti-trypsin, CD3+ve lymphocytes (total T), CD4+ve lymphocytes (helper/inducer T), CD8+ lymphocytes (cytotoxic T) and CD16+ve (natural killer cells).	Serum (serum proteins) Blood mononuclear cells Two blood samples (between 9 and 10 am)	Reported grief was negatively correlated with the number of CD16+ T cells and alpha-2 macroglobulin levels. The duration of grief was negatively correlated with the number of CD8+ T cells (*p* < 0.04) and serum levels of IgG (*p* < 0.05) and transferrin (*p* < 0.05).
Schultze-Florey et al. [52]	2012	USA	Good	Case-control study	Bereaved spouses vs. non-bereaved married/partnered	Spouse or partner	in the past 2 years (mean: 23.75 months; range 2–69 months)	N = 64; Bereaved N = 36, among which N = 13 satisfying diagnostic threshold for CG; Non-bereaved N = 28	Bereaved = 72.9 (SD = 5.8); Non-Bereaved = 72.4 (SD = 4.2)	ICG	IL-6 IL-1RA sTNFRII	Plasma	Circulating levels of IL-1RA and IL-6 were significantly higher in the bereaved group compared to non-bereaved controls. No differences in plasma cytokine levels were found between the two subgroups of bereaved individuals (CG and Non-CG groups).
Fagundes et al. [53]	2019	USA	Fair	Randomized clinical trial	Bereaved spouses	Spouse (married for at least 3 years)	Up to 14 weeks after loss	N = 99; Females N = 71 (72%); Males N = 28 (28%)	68.61 (SD = 10.7)	ICG	IFN-γ IL-6 TNF-α IL17-A IL-2.	Supernatants from mitogen-activated whole blood cell cultures	Individuals experiencing greater grief and severe symptomatology had higher levels of the proinflammatory cytokines IFN-γ, IL-6, and TNF-α than those experiencing less grief. Subjects with higher depression scores had higher levels of proinflammatory cytokines.
Bui et al. [54]	2019	USA	Good	Case-control study	Bereaved adults with a primary diagnosis of CG (Group 1) and a primary diagnosis of MDD (Group 2); psychiatrically healthy bereaved controls (Group 3)	Parent; spouse; other	//	N = 139; Group 1 N = 47; Group 2 N = 46; Group 3 N = 46 Females N = 97 (69.8%); Males N = 42 (30.2%)	Group 1 49.49 (SD = 12.87); Group 2 49.33 (SD = 13.27); Group 3 48.65 (SD = 12.7)	ICG SCI-CG	Oxytocin	Plasma	Patients with primary CG had significantly higher oxytocin levels than those with primary MDD, but not compared to bereaved controls. Using regression models, a primary or probable CG diagnosis was associated with significantly higher oxytocin levels.
Harfmann et al. [55]	2020	USA	Good	Cross-sectional study	Grievers (N = 44) vs. HC (N = 17). Grievers further divided into high grief (ICG score > 30) vs. low grief (ICG score < 30) groups	59% lost spouse/partner or child; 32% lost a parent or sibling.	Mean 160 ± 91.6 days (~5 months).	N = 61; Grievers N = 44 Controls N = 17	Grievers: 65.8 ± 9.2 Controls: 71.4 ± 7.9.	ICG	Endocannabinoids: N-arachidonoylethanolamide (AEA) and 2-arachidonoylglycerol (2-AG).	Serum, fasting morning blood draw (7–11 a.m.).	Grievers showed significantly higher AEA than healthy controls, but no difference in 2-AG. AEA levels were positively correlated with the severity of depression and anxiety symptoms, but only in grievers belonging to the low-grief group. No association was found for 2-AG.
Kang et al. [56]	2021	USA	Good	Repeated cross-sectional study	Adults aged ≥50 years divided in two groups: Grief and HC. Grief group divided in two subgroups: Grief with High loneliness (Grief-HL Group) and Low loneliness (Grief-LL Group)	Grief-HLgroup: Spouse 33%; son 22%; parent 39%; other 1% Grief-HL group: Spouse 62%; son 19%; parent 8%; other 12%	Up to 13 months before enrollment	N = 64; Grief-HL Group: N = 18; grief LL Group: N = 26; HC: N = 20 Females N = 49 (76.5%); Males N = 15 (23.5%)	Grief-HL group: 60.5 (SD = 7.5); Grief-LL group: 70.5 years (SD = 9.7); HC 70.6 (SD = 9.0)	ICG (Baseline clinical assessment and at weeks 8, 16 and 26)	Endocannabinoids: N-arachidonoylethanolamine (AEA) and 2-arachidonoylglycerol (2-AG) and cortisol (within 20.5 ± 13 days of the baseline clinical visit)	Serum	Serum AEA concentrations were significantly increased in the Grief-HL group compared with HC, but not between the two Grief groups. Serum 2-AG and cortisol concentrations did not differ among the three groups.
Brown et al. [57]	2022	USA	Fair	Cross-sectional study	Bereaved spouses divided in two subgroups: High-Grief Group (ICG score > 25); Low-Grief Group (ICG score < 25)	Spouse	Approximately 4 months after loss	N = 111; High-grief group N = 38; Low-grief group N = 73 Females N = 72 (65%); Males N = 39 (35%)	High-grief group: 66.29 (SD = 9.94); Low-grief group: 69.04 (SD = 9.04)	ICG	Interleukin-6 (IL-6)	Serum	Subjects with high grief symptoms experienced a 45% increase in IL-6 levels per hour, while those in the low grief group showed only a 26% increase. Thus, high grief was associated with a 19% greater increase in IL-6 per hour than low grief.

CG: complicated grief; SCI-CG: Structured Clinical Interview for Complicated Grief; HC: healthy controls; Non-CG: non-complicated grief; ICG: Interview for Complicated Grief; MDD: Major Depressive Disorder; IgG: immunoglobulins G; IgA: immunoglobulins A; IgM: immunoglobulins M.

## Data Availability

No new data were created or analyzed in this study. Data sharing is not applicable to this article.
